# A sweet orange mutant impaired in carotenoid biosynthesis and reduced ABA levels results in altered molecular responses along peel ripening

**DOI:** 10.1038/s41598-019-46365-8

**Published:** 2019-07-08

**Authors:** Paco Romero, María Teresa Lafuente, María Jesús Rodrigo

**Affiliations:** Department of Food Biotechnology, Institute of Agrochemistry and Food Technology (IATA-CSIC), Agustin Escardino, 7, 46980 Paterna, Valencia Spain

**Keywords:** Plant hormones, Plant molecular biology

## Abstract

Citrus fruit ripening is a complex process involving biochemical, physiological and molecular events that differ between the flesh and the peel of the fruit. We characterized sweet orange peel maturation by means of a comparative transcriptomic analysis between Navelate orange (*Citrus sinensis* L. Osbeck) and its mutant fruit Pinalate, which presents a severe blockage at early steps of the carotenoid biosynthetic pathway and consequently reduced ABA levels. Peel ripening involved the decrease of the photosynthetic activity and the transmembrane transport processes, as well as the buildup of starch and cuticular waxes and the cell wall modification. In addition, a number of biotic and abiotic stress responses, including the defense response, and the response to blue light, water deprivation and abscisic acid stimulus were modulated in a ripening-stage specific manner. The regulation of energy-related processes and secondary metabolism pathways was attenuated in Pinalate, while the molecular mechanisms underlying stress responses displayed dependency on ABA levels. These results indicate that ABA is a key signal inducing stress responses along orange peel ripening, which might determine the fruit postharvest performance.

## Introduction

Fruits from the genus *Citrus* are of main relevance worldwide because they are grown in more than 130 countries, covering more than 9.5 million ha harvested area and with an estimated production over 125 million Tm per year (FAOSTAT, 2016).

Biochemical and physiological events involved in citrus fruit development and ripening have been extensively studied for decades. Citrus fruit growth and development follows three stages: first, a cell division phase; then, a cell expansion stage of major increase in size and weight; and the third phase during which fruit growth bottoms down and most of the characteristic events of fruit maturation or ripening takes place^1–3^. Among these changes, the main affected processes include the increase in sugars and other soluble solids, and the reduction of organic acids^2^. Peel pigmentation develops as a result of chlorophyll degradation and activation of carotenoid biosynthesis, while the increase of specific volatile compounds, and an exhaustive modification of the cell wall metabolism occurs^4,5^. However, defining the criteria to uniformly determine citrus fruit maturation may not be simple because physiological changes and metabolic processes occurring in peel and pulp during maturation are autonomous and independent, mainly due to the lack of vascular connections between both tissues^6^. In spite of flavedo (outer colored cell layers of the fruit peel) is the first tissue of citrus fruit interacting with the environment and the first protection barrier of the fruit against biotic and abiotic stresses, most of the studies have been focused on the molecular processes underlying flesh maturation, and a limited number of reports investigated the molecular changes occurring along peel ripening, being most of them centered on changes in pigmentation^7–12^.

Citrus are defined as non-climacteric fruit, since respiration rate declines progressively during fruit development and full ripen fruit produce a very low and constant amount of ethylene. In contrast to the well-known role of ethylene controlling most of the maturation processes in climacteric fruits^13^, reports pointing to abscisic acid (ABA) as a major regulator of non-climacteric fruit ripening are accumulating in last years. ABA biosynthesis and the expression of the components of the ABA-signalosome have correlated with the progress of fruit ripening in strawberry, grape and sweet orange, among others^14–17^. In the peel of citrus fruits, the ABA content increases during maturation and the expression of the ABA perception system components is dependent on the hormone accumulation profile in this tissue^16,18–21^. It has been also demonstrated that alteration of the ABA perception system modifies sugar accumulation and sugar-responsive genes along strawberry fruit ripening^15^. In contrast, ABA regulated anthocyanin accumulation and cell wall modification in bilberry, but sugars appeared to play a less relevant role modulating ripening-related processes in this fruit^22^. In addition, Koyama *et al*.^14^ found that exogenous ABA application induced pigmentation in the skin of Cabernet Sauvignon grape berries stimulating anthocyanin and flavonols biosynthesis. In citrus fruit conflicting information has been reported regarding ABA role on peel fruit pigmentation. Rehman *et al*.^23^ showed that pre-harvest ABA spray regulates fruit color development in Navel orange, whose pigmentation is due to carotenoids, suggesting the involvement of ABA in the regulation of ripening-related processes. However, in Valencia sweet oranges the ABA level in flavedo increases as color develops but ABA did not trigger color break^24^. On the other hand, it has been suggested that ABA would enhance tissue sensitivity to ethylene, whereas exogenous ethylene application stimulates ABA accumulation and expression of the corresponding biosynthesis genes^18,25,26^, indicating that ABA takes part of a complex hormonal interaction in the regulation of non-climacteric fruit maturation.

Comparative transcriptomic analyses between genotypes with differential performance along maturation or responsiveness to ABA applications have proven to be efficient approaches to unravel the molecular mechanisms underlying developmental events in citrus fruits^8,9,12,27–32^. In citrus, spontaneous bud mutants occur frequently and are a valuable source of variability of important phenotypic traits. These mutants provide a promising platform to investigate the regulatory network of citrus fruit development and ripening, as demonstrated by comparative studies of spontaneous late-ripening and stay-green mutants with their parental cultivars^7,8,10,12,29,33–36^. In the context of this study, it has been described a spontaneous fruit-specific mutant, named Pinalate, derived from Navelate sweet orange^19,37^. Pinalate has a fruit-specific biochemical blockage upstream of the carotenoid biosynthetic pathway, at the level of ζ-carotene desaturation, resulting in fully ripe yellow-pigmented fruit^19^ (Rodrigo *et al*. personal communication). Moreover, the level of the β,β-xanthophyll 9-*Z*-violaxanthin, which is the main carotenoid and the precursor of ABA in ripe sweet orange fruit, is highly reduced in Pinalate fruit tissues^19,38^. Consequently, Pinalate presents reduced ABA levels in the peel along maturation as compared to its parental Navelate. In contrast to mutants presenting delayed fruit ripening, Pinalate fruit reach the same maturity index (TSS/acidity) than its parental Navelate at the end of the harvesting season. Therefore, Pinalate represents an exceptional experimental system to understand the molecular events governing peel ripening and how alteration in carotenoid biosynthesis resulting in reduced ABA levels may interfere in their regulation. In this work, special attention has been paid on how ABA might regulate stress responses along fruit maturation, even before fruit exposure to the stress.

## Results

### Comparative transcriptomic profile along peel ripening between Navelate and its Pinalate mutant

**Figure 1:**
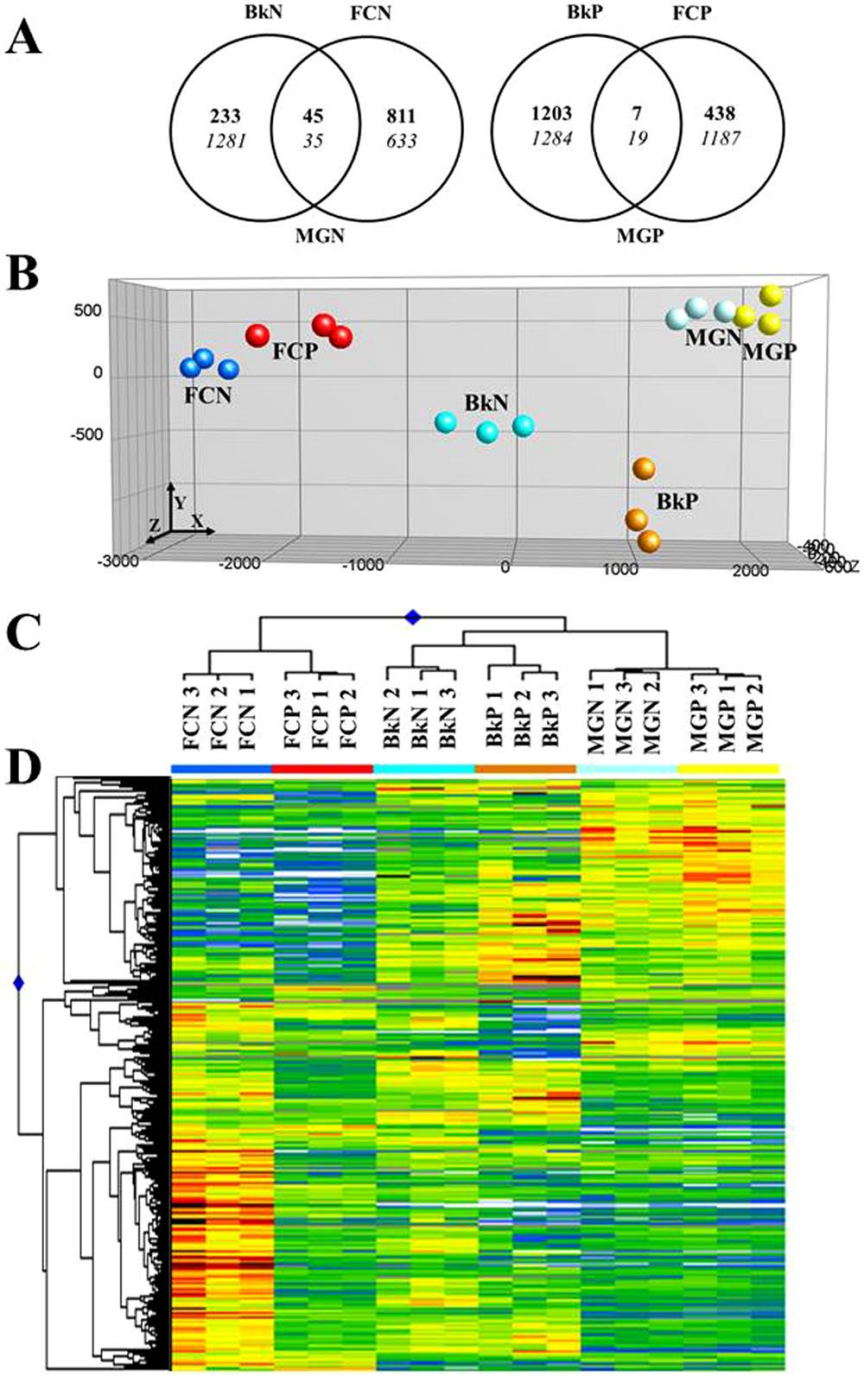
Transcriptomic comparative analyses of Navelate and Pinalate fruit along ripening. (**A**) Venn diagrams showing the distribution of differentially expressed genes (DEG, SAM analysis, FDR < 0.01) for the comparisons between breaker (Bk) and full colored (FC) Navelate (N) and Pinalate (P) fruit respect to their mature green (MG) developmental stage. Inductions and repression are indicated in bold and italics, respectively. (**B**) Principal Component Analysis (PCA), (**C**) Hierarchical Cluster Analysis (HCA) and (**D**) Heatmap large-scale transcriptional profiles based on the 1116 DEG satisfying a P < 0.001 in an ANOVA test for all conditions represented in Venn diagrams (**A**). The colors in PCA for each condition are consistent with those in HCA. The three axes in PCA account for 82.1% of the total variance among genotypes and developmental stages. Heatmap colors vary from light blue (repression) to dark red (induction). Three biological replicates from each condition were used for the analyses.

Physiological and biochemical data suggest that Breaker (Bk) stage is a turning point in the regulation of ordinary sweet orange ripening not only because of the peel color change but also because the sharp increase in ABA content and the later increase in maturity index (Supplementary Fig. S1). Fruit of Navelate and Pinalate at Bk and Full Colored (FC) stages were selected to compare changes in transcriptomic profiles with those occurring at Mature Green (MG) stage in order to identify the molecular mechanisms associated with citrus peel ripening. Venn diagrams in Fig. 1A indicated that major changes in the number of differentially expressed genes (DEG, FDR < 0.01) were found at Bk in both cultivars, being this number higher in Pinalate fruit (2513 respect to 1594). In both genotypes, Bk and FC stages shared a low number of DEG, which indicates that molecular changes involved in citrus peel ripening are regulated in a stage-specific manner. Interestingly, at the color change (Bk) the number of induced genes in the mutant was about 4-fold higher than in the parental (1210 respect to 278), whereas that of down-regulated genes between genotypes was similar. On the contrary, at FC stage the number of repressed genes in mutant fruit almost doubled that of the parental (1206 respect to 668) while induced genes were half of those found in Navelate (445 respect to 856) (Fig. 1A).

Principal Component (PCA) and Hierarchical Cluster Analyses (HCA) were performed to validate the repeatability of the microarray data across replications and to cluster samples according to their global gene expression profiles. Under all conditions, the transcriptional profiles of the three separate RNA replicate samples were tightly clustered together (Fig. 1B). The reliability of the transcriptomic results was further confirmed by comparing microarray expression values with those obtained by real-time quantitative PCR on a list of selected genes (Supplementary Table S1, Fig. S2). In addition, PCA revealed marked differences in gene expression patterns among the selected ripening stages (*X*-axis explaining the 53.5% of the total variation). Differences between genotypes were lower at FC and MG stages but remarkable at Bk stage (*Y*- and *Z*-axes explaining a 28.6% of the total variation). Accordingly, HCA revealed that samples were grouped in a ripening stage- and genotype-dependent manner (Fig. 1C). The gene-clustering in the heatmap analysis indicated that DEG responsible for the separation of genotypes and developmental stages in the PCA may be divided into two main branches: The first cluster grouped genes decreasing transcript levels from MG to FC stage, and the second is composed of genes whose expression is induced as ripening progresses (Fig. 1D). Heatmap also revealed similarity between cultivars at MG stage and substantial differences in the expression patterns of Navelate and Pinalate Bk fruit (Fig. 1D). Some clusters of genes showed high inductions in Pinalate but repression in Navelate Bk fruit, while others were repressed in both cultivars but more intensely in the mutant (Fig. 1D). At FC stage, those genes whose expression was repressed as ripening progressed showed similar transcript levels in both genotypes while those experiencing induction along maturation in the parental were still mostly repressed in the mutant at the full mature stage. Notably, some of those genes repressed in Pinalate were highly induced in the FC parental fruit (Fig. 1D).

### Alterations in peel ripening-related events in Pinalate fruit as compared to its parental Navelate

Functional categorization analysis on the DEG identified biological processes, molecular functions, cellular components and KEGG pathways significantly (P < 0.05) under- or over-represented along peel maturation in the parental and its mutant. Gene ontology analyses revealed that the high number of DEG in Navelate and Pinalate Bk fruit were only enriched in the chlorophyll binding molecular function and in the fructose and mannose metabolic pathway, both repressed in either genotype (Table 1). The number of DEG at Bk in both cultivars suggests that a large number of molecular events are being regulated at this stage, while GO analysis indicates that they are probably involved in a wide range of responses since they are not grouped in specific biological processes. At FC stage, repressed genes in the flavedo of Navelate fruit were related to photosynthesis and syncytium formation, while those induced were enriched in the response to water deprivation and to fungus, and in the metabolism of glucan (Table 1). In contrast, down-regulated genes in FC Pinalate fruit were statistically enriched in photosynthesis and lipid biosynthetic processes, whereas the response to hypoxia was the only biological process over-represented in the set of induced genes. Therefore, biological processes induced in the peel of FC parental fruit were not found in the mutant at any developmental stage, and the down-regulation of the photosynthesis, the chlorophyll binding and the fructose and mannose metabolism were the only events in common between both genotypes (Table 1). In line with this, genes encoding light-harvesting chlorophyll proteins, which are part of the antenna proteins KEGG pathway and function in the thylakoid, were repressed in both genotypes (Table 1). However, the carbon fixation pathway, tightly related to the energy metabolism involving chloroplast and mitochondria cellular components, was only repressed in the mutant fruit, as well as the flavonoid biosynthetic pathway. Last, the ribonucleoprotein complex and the intracellular non-membrane-bound organelle cellular components were over-represented in the set of Navelate FC induced genes (Table 1), suggesting an enhancement of the transcriptional activity in the parental but not in the mutant fruit.

**Table 1:** Functional categorization of differentially expressed genes (FDR < 0.01) in the flavedo of Navelate (N) and Pinalate (P) fruit harvested at full colored (FC) stage with respect to mature green (MG) fruit.

Functional Category	GO Level	GO Code	Biological Process	BkN *vs* MGN	BkP *vs* MGP	FCN *vs* MGN	FCP *vs* MGP
*Biological processes*	3	0015979	Photosynthesis			↓	↓
	3	0006950	Response to stress			↑	
	3	0019748	Secondary metabolic process				↓
	3	0006949	Syncytium formation			↓	
	4	0043283	Biopolymer metabolic process				↓
	4	0006629	Lipid metabolic process				↓
	4	0001666	Response to hypoxia				↑
	4	0009415	Response to water			↑	
	5	0019684	Photosynthesis, light reaction				↓
	5	0005976	Polysaccharide metabolic process			↑	
	5	0009620	Response to fungus			↑	
	5	0009414	Response to water deprivation			↑	
	6	0008610	Lipid biosynthetic process				↓
	7	0044264	Cellular polysaccharide metabolic process			↑	
	7	0009773	Photosynthetic electron transport in photosystem I				↓
	8	0006073	Glucan metabolic process			↑	
	8	0000271	Polysaccharide biosynthetic process			↑	
	9	0009250	Glucan biosynthetic process			↑	
*Molecular functions*	4	0016168	Chlorophyll binding	↓	↓		
*Cellular components*	3	0043228	Non-membrane-bound organelle			↑	
	5	0044424	Intracellular part			↓	
	6	0030076	Light-harvesting complex			↓	↓
	6	0030529	Ribonucleoprotein complex			↑	
	7	0043232	Intracellular non-membrane-bound organelle			↑	
	8	0031984	Organelle subcompartment			↓	
	8	0009579	Thylakoid			↓	↓
	9	0009507	Chloroplast				↓
	9	0044428	Nuclear part				↓
	9	0044435	Plastid part				↓
	9	0044436	Thylakoid part			↓	↓
*KEGG pathways*		ath00051	Fructose and mannose metabolism	↓	↓		
		ath00195	Photosynthesis			↓	↓
		ath00196	Photosynthesis - antenna proteins			↓	↓
		ath00710	Carbon fixation in photosynthetic organisms				↓
		ath00941	Flavonoid biosynthesis				↓

Arrows indicate enriched biological processes (FatiGO+, P < 0.05) induced (up) or repressed (down) into each comparison. Higher GO level indicates higher specificity.

To further uncover biological processes related to peel maturation and how Pinalate mutation might interfere in their regulation, we performed a more restrictive ontology analysis, considering only those DEG responsible for the separation of genotypes and ripening stages observed in the multivariate analyses. The 1116 genes satisfying the ANOVA test (P < 0.001) were enriched in a high number of biological processes that might be classified into two different groups. The first group would include biological processes related to primary and secondary metabolism and those involved in the redox potential and energy generation. This group is composed of the lignin, wax, and starch biosynthetic processes and of the transmembrane and photosynthetic electron transport categories. Secondly, we identified 12 different biological processes that may be grouped as biotic and abiotic stress responses (Table 2). Therefore, the next subsections will delve into the molecular mechanisms included in each group separately.

**Table 2:** Functional categorization for the differentially expressed genes (ANOVA test, P < 0.001) responsible for the separation of genotypes and developmental stages in the PCA and HCA analyses.

	GO Level	GO Term (Biological Processes)	GO Code	*p value*
Group 1		Lignin Biosynthesis		
	6	Aromatic compound biosynthetic process	0019438	0.0082
	6	Cellular amino acid derivative biosynthetic process	0042398	0.0070
	6	Phenylpropanoid metabolic process	0009698	0.0037
	8	Lignin biosynthetic process	0009809	0.0038
		Wax Biosynthesis		
	5	Lipid biosynthetic process	0008610	0.0062
	8	Fatty acid metabolic process	0006631	0.0011
	10	Wax biosynthetic process	0010025	0.0055
		Starch Biosynthesis		
	7	Cellular polysaccharide biosynthetic process	0033692	0.0068
	8	Glucan biosynthetic process	0009250	0.0013
	9	Starch biosynthetic process	0019252	0.0076
		Photosynthetic Electron Transport		
	4	Generation of precursor metabolites and energy	0006091	0.0061
	7	Photosynthetic electron transport in photosystem I	0009773	0.0038
		Transmembrane Transport		
	6	Transmembrane transport	0055085	0.0065
Group 2		Stress Responses		
	4	Aging	0007568	0.0013
	4	Response to osmotic stress	0006970	0.0071
	4	Response to oxidative stress	0006979	0.0013
	4	Response to wounding	0009611	0.0003
	5	Innate immune response	0045087	0.0047
	5	Response to cold	0009409	0.0000
	5	Response to hormone stimulus	0009725	0.0030
	5	Response to water deprivation	0009414	0.0001
	6	Defense response to fungus	0050832	0.0002
	6	Response to abscisic acid stimulus	0009737	0.0001
	6	Response to blue light	0009637	0.0083
	6	Response to chitin	0010200	0.0003

Higher GO level indicates higher specificity.

### Effects of carotenoid biosynthetic blockage and reduced ABA levels on the regulation of metabolism-related pathways, redox potential and energy generation along peel ripening

To get a deeper insight into how Pinalate mutation interferes with the regulation of the molecular mechanisms controlling the lignin, wax, starch and lipid biosynthetic processes and the transmembrane and photosynthetic electron transport along fruit ripening, genes included in the most specific biological processes for each category were studied.

Belonging to the lignin biosynthesis category, we identified the *HYDROXYCINNAMOYL-COA TRANSFERASE* (*HCT*), *CUMARATE-3-HYDROXYLASE* (*C3H*), *CINNAMOYL-COA REDUCTASE* (*CCR2*), *O-METHYLTRANSFERASE1* (*OMT1*) and *FERULIC ACID 5-HYDROXYLASE1* (*F5H*) genes. Only minor differences between genotypes were found among the expression values of these genes. In fact, *OMT1* and *F5H*, acting downstream in the pathway, were repressed in both cultivars and no significant differences were observed when compared Navelate and Pinalate FC fruit (Table 3). However, *HCT* and *C3H*, encoding the enzymes that catalyze the three first steps of the lignin biosynthetic pathway and hence regulate the accumulation of caffeic and coumaric acids, displayed opposite expression patterns during ripening in both genotypes and higher transcript levels in the parental than in the mutant at FC stage (Table 3). Similarly, the expression values of the genes belonging to the wax biosynthetic process barely differed between Navelate and Pinalate FC fruit. Thus, among the *LONG-CHAIN ACYL-COA SYNTHETASE2* (*LACS2*), *3-KETOACYL-COA SYNTHETASE6* (*KCS6*), *3-KETOACYL-COA SYNTHETASE1* (*KCS1*) and *ECERIFERUM1* (*CER1*) genes identified in this category, only *KCS1*, which participates in the catalysis of several steps for the formation of very long chain fatty acids, showed opposite regulation between genotypes and a two-fold induction in Navelate compared to Pinalate FC fruit (Table 3). Genes belonging to the starch biosynthetic process encoded for proteins with ADP-glucose pyrophosphorylase (*APL1*, *APL2*, *APL4*) and starch branching (*SBE2.1*, *SBE2.1*) activities, involved in the production of ADP-alpha-glucose and in the formation of branches in amylopectin and alpha-amylose, respectively, were induced in the parental but not in the mutant FC fruit (Table 3). On the other hand, all genes included in the photosynthetic electron transport process were repressed in Pinalate as compared to Navelate fruit (Table 3). These genes included two subunits of the photosystem I (*PSI-G*, *PSI-P*), the delta subunit of the ATP synthase (*ATPD*) and two proton pumps (*PGR5*, *PGRL1B*) involved in the electron flow in the photosystem I (Table 3). These lacks of induction and enhanced repressions in the mutant as compared to parental fruit were also found in the regulation of the transmembrane transport-related genes. Thus, Pinalate fruit was unable to induce the expression of *MULTI-ANTIMICROBIAL EXTRUSION* (*MATE*), *ABC TRANSPORTER39* (*ABCG39*) or *VACUOLAR ATP SYNTHASE F* and *H* (*AHA-F*, *AHA-H*) genes, and showed higher repression values than its parental for the *ABC TRANSPORTER32* (*ABCG32*) and a *PUTATIVE SUGAR TRANSPORTER 1* (*ESL1*) (Table 3).

**Table 3:** Genes belonging to the most specific biological processes included in selected categories from Table 2.

Citrus unigene (CFGP DB)	Homologous in *A. thaliana*	Most similar protein	Log_2_ (FCN/MGN)	Log_2_ (FCP/MGP)	Log_2_ (FCP/FCN)
**Lignin biosynthetic process (GO:0009809; Level 8)**					
*aCL4633Contig1*	*AT5G48930*	HCT (HYDROXYCINNAMOYL-COA TRANSFERASE)	0.58	**−1.62**	**−2.27**
*aCL627Contig1*	*AT2G40890*	C3H (CUMARATE-3-HYDROXYLASE)	0.47	−0.74	**−1.34**
*aCL84Contig1*	*AT1G80820*	CCR2 (CINNAMOYL COA REDUCTASE)	**2.27**	**2.14**	−1.03
*aCL38Contig8*	*AT5G54160*	OMT1 (O-METHYLTRANSFERASE 1)	**−5.45**	**−6.74**	−0.56
*aCL1203Contig1*	*AT4G36220*	F5H (FERULIC ACID 5-HYDROXYLASE 1)	**−1.95**	**−1.44**	0.69
**Wax biosynthetic process (GO:0010025; Level 10)**					
*aCL2475Contig1*	*AT1G49430*	LACS2 (LONG-CHAIN ACYL-COA SYNTHETASE 2)	**−2.22**	**−2.29**	0.32
*aCL2743Contig1*	*AT1G68530*	KCS6 (3-KETOACYL-COA SYNTHASE 6)	**−1.91**	**−1.15**	1.27
*aCL5151Contig1*	*AT1G01120*	KCS1 (3-KETOACYL-COA SYNTHASE 1)	1.75	−0.50	**−2.08**
*aKN0AAQ13YH02RM1_c*	*AT1G02205*	CER1 (ECERIFERUM 1)	**2.78**	**2.94**	−0.06
**Starch biosynthetic process (GO:0019252; Level 9)**					
*aCL9143Contig1*	*AT2G21590*	APL4 (ADP GLUCOSE PYROPHOSPHORYLASE 4)	**1.79**	1.11	**−0.75**
*aC31708E02EF_c*	*AT2G36390*	SBE2.1 (STARCH BRANCHING ENZYME 2.1)	**1.60**	0.38	**−1.06**
*aC02008F01SK_c*	*AT5G03650*	SBE2.2 (STARCH BRANCHING ENZYME 2.2)	**1.50**	0.71	**−0.75**
*aCL5827Contig1*	*AT5G48300*	APL1 (ADP GLUCOSE PYROPHOSPHORYLASE 1)	**1.46**	0.08	**−1.45**
*aCL4555Contig1*	*AT1G27680*	APL2 (ADP GLUCOSE PYROPHOSPHORYLASE 2)	**0.92**	0.09	**−0.68**
**Photosynthetic electron transport in photosystem I (GO:0009773; Level 7)**					
*aC31502D09EF_c*	*AT4G11960*	PGRL1B (PGR5-Like B)	−0.54	**−3.95**	**−2.48**
*aCL223Contig1*	*AT1G55670*	PSI-G (PHOTOSYSTEM I SUBUNIT G)	−1.25	**−3.88**	**−2.08**
*aCL5188Contig1*	*AT2G46820*	PSI-P (PHOTOSYSTEM I P SUBUNIT)	−1.68	**−2.95**	**−0.49**
*aCL2620Contig1*	*AT4G09650*	ATPD (ATP SYNTHASE DELTA-SUBUNIT)	−0.59	**−2.05**	**−1.22**
*aCL3774Contig1*	*AT2G05620*	PGR5 (PROTON GRADIENT REGULATION 5)	−0.58	**−1.90**	**−1.50**
**Transmembrane transport (GO:0055085; Level 6)**					
*aCL3641Contig1*	*AT5G17700*	MATE (MULTI-ANTIMICROBIAL EXTRUSION PROTEIN)	**1.43**	−0.57	**−2.01**
*aCL387Contig3*	*AT1G66950*	ABCG39 (ABCG TRANSPORTER 39)	**1.77**	0.24	**−1.42**
*aCL1903Contig1*	*AT4G02620*	VACUOLAR ATP SYNTHASE SUBUNIT F	**1.87**	0.75	**−1.42**
*aCL7481Contig1*	*AT3G42050*	VACUOLAR ATP SYNTHASE SUBUNIT H	**1.04**	−0.14	**−1.15**
*aC01003C11SK_c*	*AT1G08920*	ESL1 (PUTATIVE SUGAR TRANSPORTER 1)	**−1.90**	**−2.86**	−0.39
*aC08002F04SK_c*	*AT2G26910*	ABCG32 (ABCG TRANSPORTER 32)	**−1.54**	**−1.69**	0.27
*aC31803D02EF_c*	*AT5G18840*	ESL16 (PUTATIVE SUGAR TRANSPORTER 16)	0.95	**2.31**	**1.17**
**Lipid biosynthetic process (GO:0008610; Level 6)**					
*aC31301D12EF_c*	*AT2G22240*	IPS2 (INOSITOL-3-PHOSPHATE SYNTHASE2)	**−2.24**	**−3.66**	−1.15
*aC31810H11EF_c*	*AT5G49555*	PDS (PHYTOENE DEHYDROGENASE)	**−1.88**	**−3.02**	−0.88
*aKN0AAP12YO02FM1_c*	*AT5G17230*	PSY (PHYTOENE SYNTHASE)	−0.97	**−2.88**	−0.36
*aC31805F12EF_c*	*AT1G13280*	AOC4 (ALLENE OXIDE CYCLASE4)	−1.14	**−2.51**	−0.48
*aCL2475Contig1*	*AT1G49430*	LACS2 (LONG-CHAIN ACYL-COA SYNTHETASE2)	**−2.22**	**−2.29**	0.32
*aCL4449Contig1*	*AT1G19640*	JMT (JASMONIC ACID CARBOXYL METHYLTRANSFERASE)	−2.16	**−2.05**	1.19
*aCL2450Contig1*	*AT3G25820*	TPS23 (TERPENE SYNTHASE23)	−0.05	**−2.05**	−0.92
*aCL4874Contig1*	*AT5G23960*	TPS21 (TERPENE SYNTHASE21)	−0.69	**−2.02**	0.05
*aCL1628Contig2*	*AT5G42650*	AOS (ALLENE OXIDE SYNTHASE)	−0.27	**−1.88**	−2.07
*aCL906Contig1*	*AT4G11820*	HMGS (HYDROXYMETHYLGLUTARYL-COA SYNTHASE)	−0.78	**−1.74**	−0.18
*aCL2450Contig2*	*AT4G16740*	TPS3 (TERPENE SYNTHASE3)	−0.16	**−1.61**	0.23
*aCL2Contig12*	*AT4G34350*	HDR (1-HYDROXY-2-METHYL-BUTENYL 4-DIPHOSPHATE REDUCTASE)	−0.16	**−1.60**	−0.31
*aC05075A03SK_c*	*AT5G01220*	SQD2 (SULFOQUINOVOSYLDIACYLGLYCEROL2)	−1.52	**−1.57**	−0.22
*aCL4926Contig1*	*AT4G36810*	GGPPS1 (GERANYLGERANYL PYROPHOSPHATE SYNTHASE1)	−2.87	**−1.49**	−0.52
*aCL2418Contig1*	*AT3G11670*	DGD1 (DIGALACTOSYLDIACYLGLYCEROL SYNTHASE1)	−0.55	**−1.39**	−1.11
*aCL241Contig2*	*AT1G72520*	LOX1 (LIPOXYGENASE1)	−0.44	**−1.37**	−0.67
*aC02023C12SK_c*	*AT3G45140*	LOX2 (LIPOXYGENASE2)	−0.47	**−1.31**	−0.67
*aC31504D11EF_c*	*AT4G15560*	DXS2 (1-DEOXY-D-XYLULOSE 5-PHOSPHATE SYNTHASE2)	−0.35	**−1.29**	−0.40
*aC05075C09SK_c*	*AT4G03560*	FOU2 (FATTY ACID OXYGENATION UPREGULATED2)	−0.75	**−1.21**	−0.63
*aCL2162Contig1*	*AT2G26250*	KCS10 (3-KETOACYL-COA SYNTHASE10)	−1.53	**−1.21**	−0.13
*aC20001H03SK_c*	*AT3G45140*	LOX2 (LIPOXYGENASE2)	−0.60	**−1.15**	−0.51
*aCL2743Contig1*	*AT1G68530*	KCS6 (3-KETOACYL-COA SYNTHASE6)	**−1.91**	**−1.15**	1.27
*aCL2357Contig1*	*AT1G08510*	FATB (FATTY ACYL-ACP THIOESTERASES B)	−0.66	**−1.11**	−0.30
*aC02004F09SK_c*	*AT4G34640*	SQS1 (SQUALENE SYNTHASE1)	−0.10	**−0.82**	0.70
*aC31808E12EF_c*	*AT3G02580*	STE1 (DELTA7 STEROL C-5 DESATURASE)	0.14	**−0.72**	−0.21

Numbers in bold indicate statistically (SAM analysis, FDR < 0.01) significant inductions or repressions in the first term of the comparison.

Taking into account the partially overlapping results obtained separately after the two functional categorization analyses performed in this work (Tables 1 and 2), we considered that the lipid biosynthetic process (Table 1) must be included in the group of events related to primary and secondary metabolism, and deserved a deeper investigation because of this process is known as a canonical response of fruit ripening^3^. The lipid biosynthesis was statistically repressed only in the Pinalate mutant FC fruit as compared to its MG stage (Table 1), although the genes belonging to this process did not show differences between genotypes at FC stage (Table 3). This category included genes related to the methylerythritol phosphate (MEP) pathway such as *HYDROXYMETHYLBUTENYL DIPHOSPHATE REDUCTASE* (*HDR*), *DEOXYXYLULOSE 5-PHOSPHATE SYNTHASE2* (*DXS2*) and *GERANYLGERANYL PYROPHOSPHATE SYNTHASE1* (*GGPPS1*), and to the carotenoid metabolism such as *PHYTOENE SYNTHASE* (*PSY*) and *PHYTOENE DEHYDROGENASE* (*PDS*), genes related to wax and cutin biosynthesis such as *LACS2*, *KCS6*, *3-KETOACYL-COA SYNTHETASE10* (*KCS10*) and *SQUALENE SYNTHASE1* (*SQS1*), genes encoding terpene synthases involved in the generation of volatile compounds (*TPS23*, *TPS21* and *TPS3*), and genes involved in the catabolism of fatty acids to generate jasmonic acid such as *ALLENE OXIDASE CYCLASE4* (*AOC4*), *ALLENE OXIDASE SYNTHASE* (*AOS*), *JASMONIC ACID CARBOXYL METHYLTRANSFERASE* (*JMT*), *FATTY ACID OXYGENATION UPREGULATED2* (*FOU2*) and *LIPOXYGENASE1* and 2 (*LOX1* and *LOX2*).

### Effects of ABA on the regulation of biotic and abiotic stress responses along peel ripening

The response to biotic and abiotic stresses group included 12 different biological processes (Table 2). Among them, the response to water deprivation, the response to ABA stimulus, the defense response to fungus and the response to blue light, belonging to the most specific GO levels, were selected to assess transcriptional differences between parental and mutant FC fruit (Table 4). Notably, all the genes accounting for the responses to both biotic and abiotic stresses were significantly repressed in FC Pinalate as compared to FC Navelate fruit (Table 4). Indeed, parental FC fruit induced most of the genes belonging to these processes while mutant FC fruit was unable to carry out such inductions or even enhanced the repressions that eventually occurred in the parental fruit (Table 4).

**Table 4:** Genes belonging to the most specific biological processes included in the ‘Stress responses’ category in Table 2.

Citrus unigene (CFGP DB)	Homologous in *A. thaliana*	Most similar protein	Log_2_ (FCN/MGN)	Log_2_ (FCP/MGP)	Log_2_ (FCP/FCN)
**Response to water deprivation (GO:0009414; Level 5)**					
*aC34205B09EF_c	*AT5G05410*	DREB2A (AP2 DNA-BINDING PROTEIN)	**3.71**	−1.05	**−4.40**
*aCL35Contig5	*AT4G27410*	RD26 (RESPONSIVE TO DESICCATION 26)	**2.99**	−0.51	**−3.01**
*aCL146Contig3	*AT1G27730*	STZ (SALT TOLERANCE ZINC FINGER)	**3.05**	0.03	**−2.90**
*aCL6Contig4*	*AT4G02380*	SAG21 (SENESCENCE-ASSOCIATED GENE 21)	**2.85**	0.55	**−2.82**
*aCL693Contig1*	*AT5G67300*	MYBR1 (MYB DOMAIN PROTEIN R1)	**3.60**	**1.67**	**−2.22**
*aC07008A01SK_c*	*AT5G08120*	MBP2C (MICROTUBULE BINDING PROTEIN 2C)	**1.24**	−0.84	**−1.97**
*aCL23Contig3*	*AT1G47128*	RD21 (RESPONSIVE TO DEHYDRATION 21)	**1.46**	−0.05	**−1.80**
*aCL143Contig2*	*AT3G11410*	PP2CA (PROTEIN PHOSPHATASE 2CA)	**1.87**	0.05	**−1.69**
*aC34003F04EF_c*	*AT5G49230*	HRB1 (HYPERSENSITIVE TO RED AND BLUE)	**1.70**	0.22	**−1.62**
*aCL3774Contig1*	*AT2G05620*	PGR5 (PROTON GRADIENT REGULATION 5)	−0.58	**−1.90**	**−1.50**
*aCL920Contig2*	*AT3G63520*	CCD1 (CAROTENOID CLEAVAGE DIOXYGENASE 1)	**1.18**	−0.20	**−1.17**
*aCL2867Contig1*	*AT5G26990*	PUTATIVE DROUGHT RESPONSIVE PROTEIN	**1.39**	0.01	**−1.16**
*aCL87Contig3*	*AT1G78380*	GSTU19 (GLUTATHIONE S-TRANSFERASE TAU 19)	**1.79**	0.39	**−1.16**
*aC31706A07EF_c*	*AT5G67030*	ABA1 (ABA DEFICIENT 1)	0.65	−0.74	**−1.07**
*aCL9Contig15*	*AT2G17840*	ERD7 (EARLY-RESPONSIVE TO DEHYDRATION 7)	−0.16	**−1.24**	**−1.02**
*aC32007A12EF_c*	*AT1G15690*	AVP1 (VACUOLAR PROTON PUMP 1)	**1.10**	0.16	**−1.01**
*aCL3130Contig3*	*AT3G55530*	SDIR1 (SALT- AND DROUGHT-INDUCED RING FINGER1)	0.14	**−1.01**	**−0.94**
**Response to abscisic acid stimulus (GO:0009737; Level 6)**					
*aCL3520Contig1	*AT4G19230*	CYP707A1 (ABA 8′-HYDROXYLASE)	**3.97**	0.78	**−3.38**
*aCL35Contig5	*AT4G27410*	RD26 (RESPONSIVE TO DESICCATION 26)	**2.99**	−0.51	**−3.01**
*aCL706Contig1	*AT3G11820*	SYP121 (SYNTAXIN 121)	**2.22**	−0.30	**−2.71**
*aCL973Contig1*	*AT5G47390*	MYBST1 (MYB FAMILY PROTEIN)	**1.81**	−0.76	**−2.48**
*aC20005D02SK_c*	*AT2G38750*	ANNAT4 (ANNEXIN 4)	−1.20	**−4.17**	**−2.40**
*aCL3Contig25*	*AT5G59310*	LTP4 (LIPID TRANSFER PROTEIN 4)	**1.54**	−0.87	**−2.26**
*aCL693Contig1*	*AT5G67300*	MYB6 (MYB FAMILY PROTEIN)	**3.60**	**1.67**	**−2.22**
*aCL5289Contig1*	*AT5G59220*	PP2CA (PROTEIN PHOSPHATASE 2CA)	1.73	**−1.29**	**−2.19**
*aC31603C04EF_c*	*AT3G45640*	MPK2 (MITOGEN-ACTIVATED PROTEIN KINASE 2)	**2.35**	0.12	**−2.01**
*aCL143Contig2*	*AT3G11410*	PP2CA (PROTEIN PHOSPHATASE 2CA)	**1.87**	0.05	**−1.69**
*aC34103C02EF_c	*AT1G76180*	ERD14 (EARLY RESPONSE TO DEHYDRATION 14)	0.27	**−1.72**	**−1.64**
*aCL524Contig2*	*AT3G15210*	ERF3b (PUTATIVE ETHYLENE RESPONSIVE FACTOR 3b)	1.08	−0.82	**−1.53**
*aCL474Contig1	*AT3G19290*	ABF4 (ABRE BINDING FACTOR 4)	0.77	−0.79	**−1.47**
*aC18004B12Rv_c*	*AT4G21440*	MYB102 (MYB FAMILY PROTEIN)	**1.78**	0.23	**−1.42**
*aCL8290Contig1*	*AT2G25930*	ELF3 (EARLY FLOWERING 3)	1.48	0.38	**−1.39**
*aC31603H08EF_c*	*AT3G57530*	CPK32 (CALCIUM DEPENDENT PROTEIN KINASE 32)	0.43	**−1.44**	**−1.38**
*aCL9402Contig1	*AT2G46270*	GBF3 (G-BOX BINDING FACTOR 3)	0.74	−0.71	**−1.37**
*aCL943Contig3	*AT1G01720*	ATAF1 (NAC PROTEIN)	**1.73**	−0.05	**−1.36**
*aCL174Contig2*	*AT2G39800*	P5CS (DELTA1-PYRROLINE-5-CARBOXYLATE SYNTHASE 1)	0.22	−0.85	**−1.18**
*aCL638Contig2*	*AT4G04020*	PGL35 (PLASTOGLOBULIN 35)	1.08	−0.18	**−1.06**
*aCL5206Contig1	*AT1G44170*	ALDH3-H1 (ALDEHYDE DEHYDROGENASE 3 H1)	**1.45**	0.39	**−0.99**
**Defense response to fungus (GO:0009817; Level 6)**					
*aCL5483Contig1*	*AT3G21630*	CERK1 (CHITIN ELICITOR RECEPTOR KINASE 1)	**1.77**	0.00	**−1.64**
*aC34202B10EF_c*	*AT1G29340*	PUB17 (PLANT U-BOX 17)	**1.60**	0.08	**−1.57**
*aCL8631Contig1*	*AT2G44950*	HUB1 (HISTONE MONO-UBIQUITINATION 1)	**1.33**	0.08	**−1.35**
*aCL119Contig1*	*AT2G23620*	MES1 (METHYL ESTERASE 1)	**1.10**	−0.59	**−0.77**
**Response to blue light (GO:0009637; Level 6)**					
*aC34003F04EF_c*	*AT5G49230*	HRB1 (HYPERSENSITIVE TO RED AND BLUE)	**1.70**	0.22	**−1.62**
*aCL5Contig27*	*AT2G05100*	LHCB2.1 (LIGHT HARVESTING COMPLEX OF PHOTOSYSTEM II 2.1)	−1.60	**−4.28**	**−1.55**
*aCL1254Contig1*	*AT2G30520*	RPT2 (ROOT PHOTOTROPISM 2)	0.32	−1.08	**−1.36**
*aIC0AAA41DB04RM1_c*	*AT1G22770*	GI (GIGANTEA)	**1.08**	−0.72	**−1.30**
*aCL8935Contig1*	*AT5G38410*	RUBISCO SMALL CHAIN 3B	**1.25**	0.40	**−1.03**
*aCL397Contig1*	*AT5G01530*	LHCB4 (LIGHT HARVESTING COMPLEX OF PHOTOSYSTEM II 4)	**−4.03**	**−5.81**	**−0.68**
*aC08001B10SK_c*	*AT4G08920*	CRY1 (CRYPTOCHROME 1)	−0.71	**−1.62**	**−0.57**
*aCL93Contig2*	*AT4G10340*	LHCB5 (LIGHT HARVESTING COMPLEX OF PHOTOSYSTEM II 5)	−3.31	**−4.50**	**−0.33**

Numbers in bold indicate statistically (SAM analysis, FDR < 0.01) significant inductions or repressions in the first term of the comparison. Asterisks indicate genes chosen for multiple linear regression and RT-qPCR analysis.

**Figure 2:**
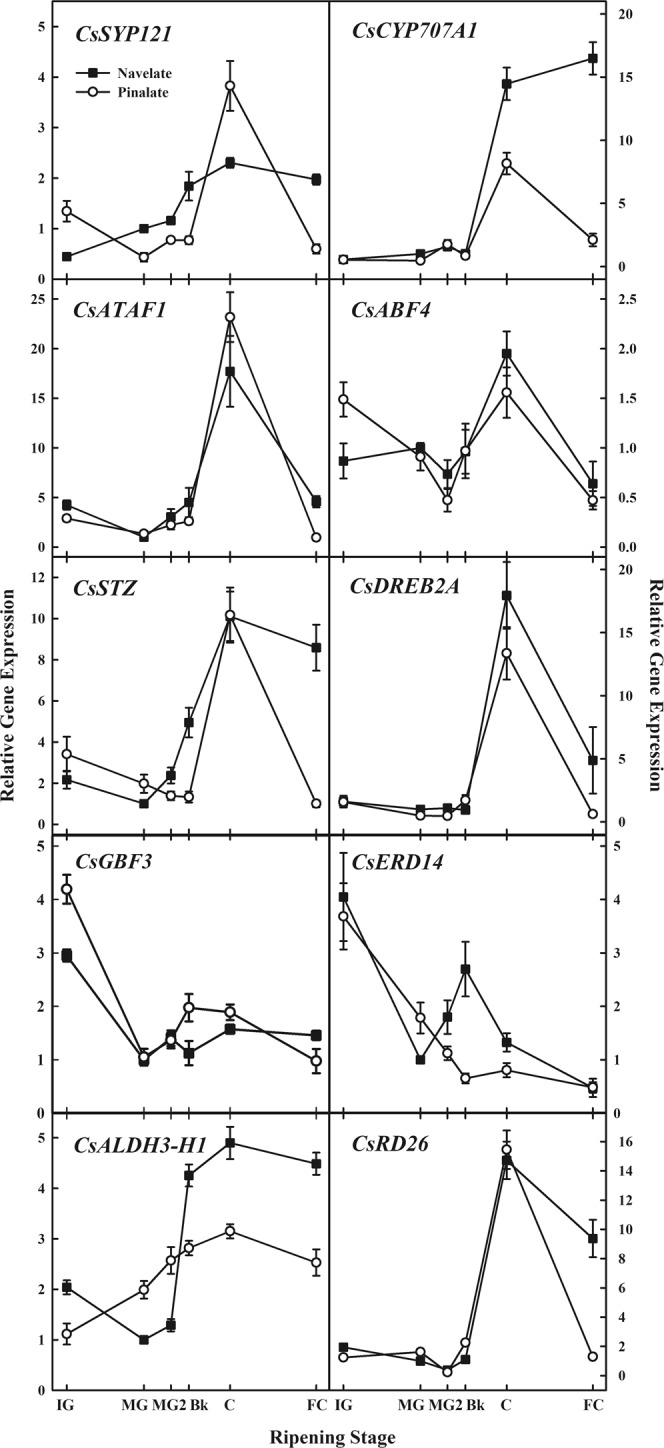
Real-time qRT-PCR expression analysis for selected genes from microarray analysis. Relative transcript abundance for selected genes belonging to ‘Response to water deprivation’ (*DREB2A*, *STZ*) and ‘Response to abscisic acid stimulus’ (*CYP707A1*, *RD26*, *SYP121*, *ERD14*, *ABF4*, *GBF3*, *ATAF1*, *ALDH3-H1*) biological processes differentially regulated in Navelate (black) and Pinalate (white) fruit harvested at six ripening stages. Transcript levels for all conditions were expressed relative to MG Navelate fruit. Data are the mean values of three biological replicates ± SE. Asterisks indicate statistical differences between genotypes according to a *t*-test (*pvalue* < 0.05) for each ripening stage.

Within the context of this work, the response to ABA stimulus merits special mention. Among genes belonging to this process we identified a gene involved in the ABA catabolism (*CYP707A1*), components of the ABA-signalosome (*PP2CA*, *CPK32* and *MPK2*), several transcription factors (*MYBST1*, *MYB6*, *ERF3b*, *ABF4*, *MYB102* and *GBF3*) and a number of loci described as ABA-responsive genes in the *Arabidopsis* model plant (*RD26*, *SYP121*, *ANNAT4*, *LTP4*, *ELF3*, *ERD14*, *ATAF1, PC5S*, *PGL35* and *ALDH3-H1*) (Table 4). We selected 8 of these genes categorized as ABA-dependent (*CsCYP707A1*, *CsSYP121*, *CsABF4*, *CsATAF1*, *CsRD26*, *CsERD14*, *CsGBF3* and *CsALDH3-H1*) and conducted their expression analysis by qRT-PCR on Navelate and Pinalate fruit harvested at 6 ripening stages in order to investigate how Pinalate mutation affects their regulation along fruit maturation (Fig. 2). To better understand this question, two genes (*CsDREB2A* and *CsSTZ*) described as ABA-independent and belonging to the response to water deprivation biological process (Table 4) were also included in the analysis. Overall, expression patterns for a high number of the genes, including those described as ABA-independent, paralleled ABA accumulation along ripening in both cultivars (Fig. 2, Supplementary Fig. S1). Thus, expression remained at low levels at green stages and sharply increased after color break to bottom down again at the end of the ripening (FC stage). Nevertheless, these genes showed higher expression values in the parental than in the mutant fruit at FC stage, when ABA levels were still 2-fold higher in Navelate than in Pinalate fruit (Fig. 2, Supplementary Fig. S1). It should be noticed that the *CsGBF3* transcription factor and the *CsERD14* dehydrin did not follow this pattern and showed the maximum expression at the most immature stage to decrease thereafter in both cultivars (Fig. 2). On the other hand, marked differences between genotypes along all ripening stages were detected for the *CsSYP121* syntaxin and the *CsALDH3* aldehyde dehydrogenase.

**Figure 3:**
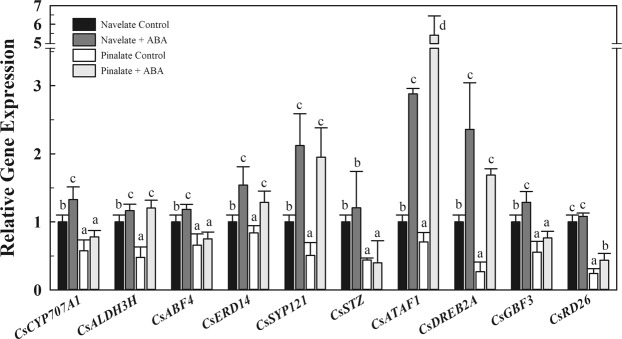
Effect of ABA treatment on the expression of stress-responsive genes. Relative transcript abundance for selected genes belonging to ‘Response to water deprivation’ (*DREB2A*, *STZ*) and ‘Response to abscisic acid stimulus’ (*CYP707A1*, *RD26*, *SYP121*, *ERD14*, *ABF4*, *GBF3*, *ATAF1*, *ALDH3-H1*) biological processes (Table 4) in Navelate and Pinalate fruit harvested at FC stage and treated or not with ABA. Transcript levels for all conditions were expressed relative to Navelate non-treated FC fruit. Data are the mean values of three biological replicates ± SE. Different letters indicate statistical differences among conditions according to an ANOVA test (*pvalue* < 0.05) for each gene.

To narrow down the participation of ABA regulating these stress-related responses, the transcriptional regulation of the same set of genes studied along ripening was assessed in Navelate and its mutant Pinalate FC fruit after applying an ABA treatment. ABA application increased the ABA levels in Navelate FC fruit by about a 3-fold (from 0.57 to 1.73 μg/g fresh weight) and about a 10-fold in the mutant FC fruit (from 0.2 to 1.93 μg/g fresh weight), which means that ABA was efficiently uptaken by the fruit and similar levels of the hormone were reached in fruit of both cultivars upon 1 week of treatment. Consistently with data reported in Fig. 2, most of the genes were statistically repressed in the mutant as compared to control (non-treated) Navelate fruit (Fig. 3). After ABA treatment, all studied genes but *CsSTZ* and *CsRD26* were induced in Navelate fruit as compared to control fruit (Fig. 3). In ABA-treated Pinalate fruit, however, *CsALDH3H*, *CsERD14*, *CsSYP121*, *CsATAF1, CsDREB2A* and *CsRD26* transcripts accumulation was increased as compared to control non-treated fruits (Fig. 3). In addition, not all these genes reached the same (or higher) transcript levels in Pinalate fruit after ABA treatment than in the parental. In fact, only *CsALDH3H*, *CsERD14*, *CsSYP121*, *CsATAF1* and *CsDREB2A* expression levels were similar between ABA-treated Navelate and Pinalate fruit; the expression of other genes such as *CsCYP707A1*, *CsABF4*, *CsSTZ*, *CsGBF3* and *CsRD26* were still reduced in ABA-treated Pinalate as compared to control Navelate fruit, highlighting the partial ability of the mutant fruit after exogenous ABA application to recover the transcript levels found in the parental fruit.

## Discussion

In order to uncover whether ABA is an important factor regulating the ripening program of citrus peel, we selected Pinalate, a spontaneous fruit-specific mutant from the sweet orange Navelate (*Citrus sinensis* L. Osbeck) displaying a blockage upstream of the carotenoid biosynthetic pathway and reduced ABA levels, to perform transcriptomic comparisons at different peel maturation stages. Under our experimental conditions, we confirmed the suitability of Pinalate for this study based on its reduced ABA accumulation in the flavedo along fruit ripening (Supplementary Fig. S1). In agreement with previous works^16,19,37^, no differences in ABA content were found between cultivars whilst fruits remained green (IG, MG and MG2) and the highest accumulation of ABA in the flavedo of parental fruit was found at Bk stage, showing more than 4-fold ABA levels than Pinalate (Supplementary Fig. S1). At the end of the experiment (FC stage), ABA levels were still 2-fold higher in the parental than in the mutant (Supplementary Fig. S1). As expected, transcriptomic analyses revealed that the expression of a high number of genes is regulated along peel ripening in a developmental stage-specific manner as has been recently reported in mandarin fruits^12^. Our results further showed that the peel of the Pinalate mutant fruit displayed an altered ripening transcriptional program compared to its parental Navelate (Fig. 1). Differences in ABA levels were consistent with these deviations since expression patterns were most similar at MG stage, when ABA content was no different between genotypes, while transcriptional profiles became more different as ABA accumulation differed, being maximum at Bk and still significant at FC stage (Fig. 1 and Supplementary Fig. S1).

Functional categorization analyses showed that the photosynthetic activity diminished as ripening progressed in Navelate and Pinalate fruit peel (Tables 1 and 2). The decline in the activity of the photosynthetic apparatus in both climacteric and non-climacteric fruit is a well-known ripening regulated process as consequence of chlorophylls degradation, disappearance of thylakoidal membranes and reorganization of plastid internal membranes as consequence of the transformation of chloroplasts into chromoplasts^38–40^. The severe blockage at the ζ-carotene level in Pinalate fruit^19^ (Rodrigo *et al*., personal communication) provokes a massive accumulation of colorless carotenes (phytoene and phytofluene) in chromoplasts of the mutant and the presence of atypical suborganellar structures^38^ which may result in altered plastid organization and enhanced repression of all those photosynthesis-related functional categories as compared to its parental fruit (Supplementary Table S2 and Table 3). The elevated accumulation of colorless carotenes might have other consequences at metabolic level since these compounds are precursors of apocarotenoids and other signaling molecules, such as strigolactones. In fact, an Arabidopsis mutant lacking ζ-carotene desaturation presents alterations in chloroplast-encoded and photosynthesis-related gene expressions resulting in abnormal development and morphology in vegetative tissue^41^. Much less information is available about how unusual accumulation of these carotenes as results of an early bottleneck in the carotenoid pathway might affect citrus fruit peel development and ripening. Another spontaneous mutant fruit from the sweet orange Navelate accumulating upstream carotenes (phytoene and phytofluene) in the flavedo^38,42^, named CaraCara, does not display an altered developmental phenotype although, in this case, ABA levels in the flavedo are very similar between the mutant and the parental fruit, and molecular analyses on this mutant along fruit maturation have been limited to pulp tissues^43^. In tomato fruit, VIGS mutants with altered ZDS (ζ-carotene desaturase) or Z-ISO (ζ-carotene isomerase) activities causing a similar chemotype to Pinalate in terms of carotenoids accumulation indicate that no other trait but color and carotenoid accumulation are affected^44^. Therefore, we cannot rule out the possibility that carotene-derived signaling molecules might participate somehow in the regulation of the ripening process, but present results still support the hypothesis that ABA might also play a role controlling photosynthesis, as previously suggested by Farnsworth^45^. In line with this, there is long compelling evidence that the biosynthesis of flavonoids, specifically repressed in Pinalate FC fruit (Table 1), is upregulated in response to photosynthetic redox unbalance in plants and that these compounds likely interact with auxin and ABA signaling^46^. Moreover, xanthophylls, which are accessory pigments in the photosynthesis and protect the photosynthetic apparatus from photooxidation^47^, are highly reduced in the Pinalate compared to parental fruit^19^. These facts might provide a scenario for the Pinalate mutant where its inability to induce the flavonoids and xanthophylls antioxidant functions, and an altered photosynthesis activity might have consequences on the energy status and redox balance of this fruit. Further support for this idea comes from the fact that transmembrane transport and sugar metabolism are impaired in Pinalate fruit. Indeed, two subunits of the vacuolar ATP synthase proton pump involved in the generation of ATP at the tonoplast, a MATE proton antiporter providing transmembrane gradient, and an ABC transporter (*ABCG39*), participating in the influx of substances through ATP consumption, were induced in Navelate but not in the mutant FC fruit. Also, the repression of a putative sugar transporter (*ESL1*) that imports glucose to the cytosol was enhanced in Pinalate mutant FC fruit (Table 3). These results suggest that ABA is involved in the fruit energy status and redox potential maintenance. This is especially interesting when considering that energy shortage might be implicated in the susceptibility of citrus fruit to develop physiological peel disorders^48^, since Pinalate is much more prone than Navelate to develop peel damage^37,49–51^ and results from this work converge in the idea of an unbalanced energy status in the mutant fruit.

In addition, despite both genotypes repressed the fructose and mannose metabolism pathway (Table 1), only Navelate induced the starch biosynthetic process (Table 1), and the induction of the genes responsible for the activation and structural conformation of starch (*APL*s and *SBE2*s, respectively) was abolished in the Pinalate mutant (Table 3). These results agree with a previous report showing that starch accumulates in citrus fruit peel along maturation^5^ and converge with other works in citrus flesh and other non-climacteric fruit considering sugar metabolism as a main response occurring along ripening^27,29,52,53^. Nevertheless, the induction of starch biosynthesis only in Navelate contrasts with the higher accumulation of starch observed in plastids of Pinalate fruit^38,54^. One explanation for this apparent discrepancy is that the starch overloading in Pinalate peel might be a result of its high accumulation of sucrose in the cytosol. In fact, non-photosynthetic cells degrade sucrose in the cytosol producing intermediates that can be imported into the amyloplast for starch synthesis; and sucrose content is much higher in Pinalate than in Navelate peel^54^. Therefore, it could be thought that Pinalate, despite the absence of induction *APL*s and *SBE*s genes, is able to accumulate more starch in the plastids than its parental Navelate because of the higher accumulation of starch precursors in the cytosol.

Regulation of lipid metabolism is a canonical response occurring along flesh ripening^3,4^ and it is well-known that some lipid compounds such as specific volatile and carotenoids increase with ripening both in flesh and citrus peel^2^. Results from the present study indicate that ABA levels might be a factor determining the accumulation of these compounds along citrus peel ripening since this process was specifically repressed in the Pinalate mutant (Table 1). Further support for this idea comes from recent reports suggesting a role for ABA in lipid metabolism along fruit ripening when specifically considering carotenoids and triterpenoid biosynthetic pathways in other fruit species^23,55–58^. It should be also mentioned that the expected upregulation of the *PSY* and *PDS* carotenoid biosynthetic genes in Navelate fruit along ripening^20,58^ was not observed in our transcriptomic data, since results did not reveal statistical differences for those genes between MG and FC stages (Table 3). It has been previously described by Kato *et al*.^20^ and Alquézar *et al*.^42^ that the expression of these genes is maximum at breaker stage and follows a decrease in transcript levels at late ripening stages (FC stage). However, since our GO analyses did not reveal significant lipid metabolism-related processes at Bk stage either in Navelate or Pinalate fruit, we selected the FC fruit for the global transcriptional analyses comparing both genotypes (Table 3). Thus, the lack of induction of the *PSY* and *PDS* carotenoid biosynthetic genes in Navelate FC fruit is consistent with the expression patterns reported by Kato *et al*.^20^ and Alquézar *et al*.^42^ and also with the reduction in ABA content observed at FC stage in the flavedo of Navelate fruit (Supplementary Fig. S1).

The regulation of cell wall metabolism, including lignin biosynthesis, has been highlighted as a major ripening-related process in a number of fruits, including citrus^2,11,13^. Our results indicate that *HCT* and *C3H* genes involved in lignin biosynthesis were upregulated in Navelate whereas *OMT1* and *F5H* genes, acting downstream in the pathway, were repressed (Table 3). This regulation would redirect the metabolic flux to the accumulation of caffeic and coumaric acids in sweet orange as peel maturation progressed, which would fit with previous reports proposing a negative role for ABA in lignin accumulation^59^. In Pinalate mutant, however, *HCT* and *C3H* are not induced, which suggests that cell wall composition would differ between cultivars at FC stage. In line with this, our results indicate that wax biosynthesis is regulated along peel maturation (Table 2), which is in concordance with results from previous investigations demonstrating the increase in wax content during the maturation of citrus fruits^11,60,61^. A recent report on tomato fruit proposed a minor participation of ABA on fruit cuticle regulation, while ABA-deficiency deeply influenced cuticle properties in leaves^62^. A role for ABA in the regulation of citrus fruit cuticle formation remains elusive, but results presented here suggest a model where the increase in cuticular wax content as fruit maturation progresses is at least partially regulated by ABA (Tables 2 and 3). Thus, the distinctive regulation of particular genes along ripening, such as the *KCS1* involved in the formation of very-long chain fatty acids, might lead to different cuticle properties and composition in Pinalate FC fruit. Cuticular wax composition and load have been associated with postharvest performance and quality attributes in fruits^63,64^, and more specifically in sweet oranges^60,61,65^. Therefore, results from the present study encourage further research comparing wax accumulation and composition in Navelate and Pinalate fruit to elucidate whether ABA is indeed involved in this process and the consequences in fruit water loss during postharvest.

Responses to biotic or abiotic stresses have been rarely pointed out as part of the fruit ripening program, and few sets of stress-related genes have been shown to be regulated in grapevine and apricot skins and in citrus flesh along ripening^29,66,67^. We identified 12 biological processes related to biotic and abiotic stresses differently regulated as peel ripening progressed in both Navelate and Pinalate fruit (Table 2). A detailed study on the expression values of the genes belonging to these categories revealed that Pinalate fruit was systematically unable to carry out the inductions occurring in the parental fruit, or even enhanced repressions when they eventually occurred in Navelate (Table 4), pointing out an important role for ABA in the regulation of stress responses in citrus fruit along ripening.

As refer to biotic stress responses, a role for ABA is not straightforward due to its multifaceted function depending on the tissue, the developmental stage, the pathogen and the plant species studied^68^. In this study, the genes involved in the defense response to fungus were induced in the parental Navelate while no change or even repressions were found in the mutant fruit (Table 4), suggesting a role for ABA in the defense response of citrus fruit^69^. In line with this, it is known that blue light increases the citrus fruit resistance to *Penicillium* decay^70,71^ and, interestingly, functional categorization revealed the induction of blue light response in Navelate but not in Pinalate FC fruit, which may develop more decay than the parental fruit during postharvest storage^37^.

On the other hand, the participation of ABA in the abiotic stress responses in plant and fruit is well recognized. In citrus, Romero *et al*.^28^ reported that ABA is a key signal in the Navelate fruit response to water stress during postharvest, although this response also included ABA-independent genes. Interestingly, both the ABA-dependent and -independent genes were induced in Navelate but not in Pinalate fruit, which suggested that Pinalate might present a defect in the whole response to dehydration^28^. In addition, Romero *et al*.^16,28,49–51^ and Lafuente *et al*.^69^ have reported under different conditions that Pinalate is not fully able to respond to ABA treatment at physiological or molecular levels and demonstrated that Pinalate, besides its deficient ABA accumulation is partially insensitive to this hormone because of the altered regulation of the ABA perception system components^16^. Here, most of the genes belonging to the response to water deprivation, including ABA-dependent and independent genes, and to the response to ABA stimulus were induced in Navelate but not in Pinalate FC fruit (Table 4 and Fig. 2). Despite Pinalate fruit is a spontaneous mutant with reduced ABA levels from Navelate rather than a knockout mutant, and the partial ABA-insensitivity of Pinalate fruit^16,69^, an ABA treatment was applied to Navelate and Pinalate fruit to narrow down the putative role of ABA in the regulation of these stress-related genes. Effectively, only some of the studied genes responded to the ABA-treatment inducing their expression in Pinalate, and not all of them recovered the mutant phenotype and reached the transcripts levels observed in the parental fruit (Fig. 3). Therefore, these results agree with a deficient ABA-signaling in Pinalate fruit and still support a main role for ABA in the regulation of stress-related responses along peel ripening in citrus fruit. In addition, our findings agree with the hypothesis of a failure of the whole water stress response in Pinalate and further suggest that responsiveness to dehydration is acquired by Navelate fruit along ripening and not only after the fruit exposure to the postharvest stress.

In summary, this study reveals that peel ripening involves the regulation of biotic and abiotic stress responses, and provides further evidence for the regulation of other canonical responses along citrus peel maturation such as the induction of sugars accumulation, the repression of the photosynthesis, changes in volatile and carotenoid profiles, the increase in wax and cutin biosynthesis and the cell wall lignification^2,3^. Interestingly, the induction of stress responses makes sense when considering that the flavedo is the first barrier between the fruit and the environment. Along peel maturation, most of the energy-related processes and secondary metabolism pathways appeared to be partially attenuated by the reduced ABA levels found in Pinalate fruit, although the participation of other carotene-derived signaling molecules in their regulation cannot be ruled out. In contrast, processes related to biotic and abiotic stress responses were dependent on the hormone content. Therefore, these results point to ABA as a key signal involved in the acquisition of responsiveness against stresses along fruit maturation, which might be relevant for determining the postharvest behavior of these fruits.

## Material and Methods

### Plant material

Fruits of Navelate (*Citrus sinensis* L. Osbeck) orange and its spontaneous fruit-specific Pinalate mutant were randomly harvested from adult trees grown in experimental orchards under normal cultural practices at Citrus Germoplasm Bank at Instituto Valenciano de Investigaciones Agrarias (Moncada, Valencia, Spain). Both parental and mutant trees are subjected to the same environmental conditions and agronomical practices. Fruit from each cultivar were harvested at different ripening stages: Immature Green (IG, harvested 135 days after bloom (DAB)), Mature Green (MG, 196 DAB), Mature Green 2 (MG2, 217 DAB), Breaker (Bk, 231 DAB), Colored (C, 263 DAB) and Full Colored (FC, 330 DAB), and immediately delivered to the laboratory. Fruit maturity index was calculated by dividing the °Brix of the extracted juice by its acid content. Peel color was expressed as the *a/b* Hunter ratio and analyzed by using a Minolta CR-300 Chromameter (Konica Minolta Inc, USA) at three locations around the equatorial plane of the fruit. For the ABA treatment assay, fruits from Navelate and Pinalate cultivars were harvested at FC stage and divided into two groups. The first group was treated with ABA (Sigma-Aldrich) by dipping the fruits for 1 min in an aqueous solution of 1 mM ABA containing 0.7% ethanol to dissolve the hormone. Control non-treated fruits (second group) were dipped into 0.7% ethanol following the same procedure. Fruits were dried at room temperature and stored for one week in chambers simulating temperature field conditions at this time of the season (12 °C) and avoiding dehydration stress (90–95% relative humidity). Flavedo samples for both the ripening and the ABA treatment assays were collected from the total surface of fruit, frozen and homogenized in liquid nitrogen, and kept at −80 °C until analysis. Three biological replicates of at least 5 fruit each were collected at each sampling period.

### RNA isolation, cDNA labeling and microarray hybridization

Total RNA was extracted from frozen flavedo samples as described in Romero *et al*.^28^. Total RNA was treated with Ribonuclease-free DNase (Ambion) following the manufacturer’s instructions for removing possible genomic DNA contaminations. For microarray purposes, total RNA (30 μg) samples from each biological replicate were labeled with the indirect method by incorporation of 5-(3-aminoallyl)-2-deoxi-UTP into single-stranded cDNA during reverse transcription. cDNA synthesis and purification, dye coupling, and labeled-cDNA purification were accomplished according to the method described by Forment *et al*.^72^. cDNA samples were Cy5-labeled and co-hybridized with a Cy3-labeled cDNA reference pool from a mixture containing equal amounts of RNA from all experimental samples assayed. Microarray hybridization and slide washes were performed as described by Romero *et al*.^28^. The cDNA microarrays used were developed in the framework of the Spanish ‘Citrus Functional Genomics Project’ (http://bioinfo.ibmcp.upv.es/genomics/cfgpDB/), and contained 21081 putative unigenes (20 K) isolated from 52 cDNA libraries of citrus from a wide range of varieties, developmental and fruit ripening stages, and from different tissues subjected to biotic and abiotic stress conditions^73^, covering more than two thirds of the *Citrus* genome.

### Transcriptomic data acquisition and analysis

Hybridized microarrays were scanned by using a GenePix 4000A scanner (Axon Instruments) equipped with GenePix Pro 6.0 image acquisition software, following manufacturer’s instructions to adjust the channels intensity ratio to 1.0 and the percentage of saturated spots close to 1%. Non-homogeneous and aberrant spots were discarded. Only spots with background–subtracted intensity greater than 2-fold the mean of background intensity were used for normalization and further analysis. In order to compensate labeling differences among samples and other non-biological sources of variability, results were normalized by using Print-Tip-Lowess method, included in the Acuity 4.0 software (Axon Instruments) by using background subtracted median values and an intensity-based Lowess function within and among microarrays. Thereafter, differentially expressed genes for all possible pairwise comparisons were determined by applying the Significant Analysis of Microarrays (SAM) program from the TM4 Microarray Software Suite. Genes that satisfied a statistical threshold (FDR, False Discovery Rate) lower than 0.01 were identified as differentially expressed genes (DEG) and represented in Venn diagrams. Principal Component (PCA) and Hierarchical Cluster (HCA) Analyses, and the associated expression Heatmap were performed by using the GeneMaths XT software package (Applied Maths; http://www.applied-maths.com). ANOVA test (Benjamini-Hochberg FDR < 0.001) and Pearson’s product-moment correlation coefficient were used to identify DEG and to measure gene-to-gene correlation, respectively. FatiGO+ tool (Babelomics, http://bioinfo.cipf.es) was used to identify biological processes, molecular function, cellular components and KEGG metabolic pathways significantly under- or over-represented in a set of DEG relative to a reference group containing all genes present in the microarrays. In these analyses, a Fisher two tailed test with a *p-*value lower than 0.05 was applied and the specificity of the process increases with the GO level from 3 to 10.

### RT-qPCR expression analysis

Reverse transcription followed by quantitative polymerase chain reaction analysis was performed to validate microarray results and to examine the time-course expression pattern of selected genes along fruit ripening or in response to ABA treatment as described by Romero *et al*.^16^. Forward (F) and reverse (R) sequences for specific primers are shown in Supplementary Table S1. Three reference genes (*CsACT, CsGAPDH* and *CsTUB*) were used for data normalization. Statistical analysis was carried out by using the Relative Expression Software Tool (REST, http://rest.gene-quantification.info). Each sample was analyzed in triplicate and mean ratios ± SE were calculated.

### ABA analysis

The ABA was extracted from 1 g fresh weight frozen flavedo with 80% acetone containing 0.5 g L^−1^ citric acid and 100 mg L^−1^ of butylated hydroxytoluene as previously described by Lafuente *et al*.^18^. After centrifugation the supernatant was diluted in 3 serial dilutions in ice-cold TBS (6.05 g Tris, 8.8 g L^−1^ NaCl and 0.2 mg L^−1^ Mg Cl_2_ at pH 7.8) and 3 samples for each dilution were analyzed by indirect ELISA. The results are the means of three replicate samples ± SE.

### Statistical analysis

All values are given as the mean of the three replicate samples ± SE, and a mean comparison using the Tukey’s test was performed to determine significant differences (P ≤ 0.05). The analyses were performed with the Statgraphics Plus 4.0 Software (Manugistics, Inc.).

## Supplementary information

Supplementary Information

## Data Availability

All data generated or analyzed during this study are included in this published article (and its Supplementary Information Files).
