# Supplementary material for: A sweet orange mutant impaired in carotenoid biosynthesis and reduced ABA levels results in altered molecular responses along peel ripening
Source: Sci Rep. 2019 Jul 8;9:9813. doi: 10.1038/s41598-019-46365-8 (PMC6614452; doi:10.1038/s41598-019-46365-8)

A sweet orange mutant impaired in carotenoid biosynthesis and reduced ABA levels results in altered molecular responses along peel ripening

Paco Romero, María Teresa Lafuente and María Jesús Rodrigo*

Supplementary Information

Table S1. Selected genes and primers used for qRT-PCR analysis.

Gene	Citrus unigene (CFGP DB)	Most similar protein	Homologous in *A. thaliana*	Forward / Reverse	Sequence 5' → 3'	Amplicon size (bp)
CsABF4	*aCL474Contig1*	ABRE binding factor 4	*AT3G19290*	F	GCTGGAGAATCAAATGCTCCT	216
				R	TGCCCTCACCAAGAACTCCTC	
CsALDH3H1	*aCL5206Contig1*	Aldehyde Dehydrogenase 3 H1	*AT1G44170*	F	GGAGTGAGGAGGAGACGAAGA	188
				R	ACGAAGAGCGTCGACGATGTC	
CsATAF1	*aCL943Contig3*	NAC protein	*AT1G01720*	F	CCCAAATGGAATGATCTAGGC	202
				R	GGACCTAGCATACTACGATGT	
CsCYP707A1	*aCL3520Contig1*	ABA-8'-hydroxylase	*AT4G19230*	F	GGCTGGCCTTATATTGGAGAA	204
				R	TTTGCTAGCTGGAAATGTTGG	
CsDREB2A	*aC34205B09EF_c*	AP2-domain DNA-binding protein	*AT5G05410*	F	CGGAGAATGGACGGTGTGATT	254
				R	CAGACTGGTTGATGACGTCGT	
CsERD14	*aC34103C02EF_c*	Early response to dehydration 14	*AT1G76180*	F	GCCATGAGTACGAGCCGAGTG	251
				R	AGAGCTGCTGGTGGATCGGTG	
CsGBF3	*aCL9402Contig1*	G-box binding factor 3	*AT2G46270*	F	CCCGAGTTGCTATTCCGCCAT	215
				R	GGTTACAGCAGCAGGCGAGGT	
CsRD26	*aCL35Contig5*	Responsive to desiccation 26	*AT4G27410*	F	CCGAACTTCTGTAGCCTGGT	230
				R	CCCTCCCGACTCAAGATCAC	
CsSTZ	*aCL146Contig3*	Salt tolerance zinc finger	*AT1G27730*	F	GCCTTGAGCCATGGACCAAAC	172
				R	GCAGGAGACTTCTGCCGCTGT	
CsSYP121	*aCL706Contig1*	Syntaxin 121	*AT3G11820*	F	TCCTTCTCTCGCTTCCGCAGC	235
				R	GCAGGTCTTTCACGGCTTTCG	
CsACT	*aCL563Contig1*	Actin7	*AT5G09810*	F	TTAACCCCAAGGCCAACAGA	176
				R	TCCCTCATAGATTGGTACAGTAT	
CsGAPDH	*aCL715Contig1*	Glyceraldehyde-3P dehydrogenase	*AT1G13440*	F	CGTCCCTCTGCAAGATGACTCT	204
				R	GGAAGGTCAAGATCGGAATCAA	
CsTUB	*aCL2738Contig1*	Tubulin1	*AT1G75780*	F	GCATCTTGAACCCGGTAC	158
				R	ATCAATTCGGCGCCTTCAG	

Table S2. Genes belonging to the most specific photosynthesis-related functional categories included in Table 1 and Table 2. Numbers in bold indicate statistically (SAM analysis, FDR<0.01) significant inductions or repressions in the first term of the comparison.

**photosynthesis, light reaction (GO:0019684)**	**Level 5**	**BP**							
#name	Log_2 (BKN / MGN)	Log_2 (FCN / MGN)	Log_2 (FCN / BKN)	Log_2 (BKP / MGP)	Log_2 (FCP / MGP)	Log_2 (FCP / BKP)	Log_2 (MGP / MGN)	Log_2 (BKP / BKN)	Log_2 (FCP / FCN)
AT5G64040_Photosystem I reaction centre subunit N - chloroplast precursor (unigene aCL1971Contig1)	-1.05	-1.45	-0.40	-1.01	**-3.99**	**-2.98**	0.72	0.75	-1.83
AT4G11960_expressed protein (unigene aC31502D09EF_c)	0.89	-0.54	-1.43	0.56	**-3.95**	**-4.50**	0.93	0.60	**-2.48**
AT1G55670_Photosystem I reaction center subunit V - chloroplast precursor (unigene aCL223Contig1)	-0.80	-1.25	-0.45	-0.70	**-3.88**	**-3.17**	0.55	0.65	**-2.08**
AT4G12800_Photosystem I subunit XI (unigene aCL2872Contig1)	0.45	-1.42	**-1.87**	0.65	**-3.71**	**-4.35**	0.53	0.73	**-1.75**
AT1G60950_Ferredoxin - chloroplast precursor (unigene aCL6164Contig1)	0.30	-1.30	-1.60	-0.15	**-3.43**	**-3.28**	0.01	-0.45	**-2.12**
AT1G79040_Chloroplast photosystem II 10 kDa protein (unigene aCL148Contig1)	-0.16	**-1.54**	-1.38	0.28	**-3.27**	**-3.55**	0.34	0.78	**-1.39**
AT2G46820_Thylakoid membrane phosphoprotein 14 kDa - chloroplast precursor (unigene aCL5188Contig1)	-1.48	-1.68	-0.20	-0.45	**-2.95**	**-2.50**	0.79	1.82	-0.49
ATCG00280_Photosystem II 44 kDa reaction center protein precursor (unigene aKN0AAQ3YK09RM1_c)	0.63	**-1.62**	**-2.25**	-2.58	**-2.15**	0.43	0.68	-2.53	0.15
AT2G05620_Expressed protein (unigene aCL3774Contig1)	0.46	-0.58	-1.04	-1.02	**-1.90**	-0.88	-0.18	-1.66	**-1.50**
AT3G61470_Chlorophyll a-b binding protein 7 - chloroplast precursor (unigene aCL242Contig1)	-0.97	-1.27	-0.30	-0.09	**-1.82**	**-1.73**	0.29	1.17	-0.26
AT1G60600_At1g60600 (unigene aC31006G09EF_c)	0.58	0.57	-0.01	0.48	**-1.63**	**-2.12**	1.46	1.36	-0.74
AT3G11670_Digalactosyldiacylglycerol synthase 1 (unigene aCL2418Contig1)	0.12	-0.43	-0.55	0.50	**-1.39**	**-1.89**	-0.14	0.24	-1.11

**chlorophyll binding (GO:0016168)**	**Level 4**	**MF**							
#name	Log_2 (BKN / MGN)	Log_2 (FCN / MGN)	Log_2 (FCN / BKN)	Log_2 (BKP / MGP)	Log_2 (FCP / MGP)	Log_2 (FCP / BKP)	Log_2 (MGP / MGN)	Log_2 (BKP / BKN)	Log_2 (FCP / FCN)
AT5G01530_Lhcb4-protein (unigene aCL397Contig1)	-1.45	-4.03	-2.58	-1.09	**-5.81**	**-4.72**	1.10	1.46	-0.68
AT1G61520_Chlorophyll a-b binding protein 8 - chloroplast precursor (unigene aCL247Contig1)	-2.51	-3.33	-0.82	-1.23	**-4.42**	**-3.19**	0.34	1.62	**-0.75**
AT1G15820_Chlorophyll a/b-binding protein CP24 (unigene aCL1214Contig1)	-1.41	-2.74	**-1.33**	-0.73	**-3.89**	**-3.16**	0.98	1.67	**-0.17**
AT1G29910_Chlorophyll a/b-binding protein (unigene aCL14Contig7)	-0.86	-2.59	-1.73	-0.72	**-4.63**	**-3.92**	0.85	1.00	**-1.19**
AT3G47470_Chlorophyll a/b-binding protein (unigene aCL2238Contig1)	-1.91	**-2.45**	-0.54	-1.38	**-3.35**	**-1.98**	0.88	1.42	**-0.02**
ATCG00280_Photosystem II 44 kDa reaction center protein precursor (unigene aKN0AAQ3YK09RM1_c)	0.63	-1.62	-2.25	-2.58	**-2.15**	**0.43**	0.68	-2.53	0.15

**chlorophyll binding (GO:0016168)**	**Level 4**	**MF**							
#name	Log_2 (BKN / MGN)	Log_2 (FCN / MGN)	Log_2 (FCN / BKN)	Log_2 (BKP / MGP)	Log_2 (FCP / MGP)	Log_2 (FCP / BKP)	Log_2 (MGP / MGN)	Log_2 (BKP / BKN)	Log_2 (FCP / FCN)
AT5G01530_Lhcb4-protein (unigene aCL397Contig1)	-1.45	**-4.03**	-2.58	-1.09	**-5.81**	**-4.72**	1.10	1.46	-0.68
AT1G29910_Chlorophyll a/b-binding protein (unigene aCL14Contig7)	-0.86	**-2.59**	-1.73	-0.72	**-4.63**	**-3.92**	0.85	1.00	-1.19
AT4G10340_Type I (26 kD) CP29 polypeptide (unigene aCL93Contig2)	**-1.45**	-3.31	-1.85	-2.17	**-4.50**	-2.33	0.86	0.15	-0.33
AT1G61520_Chlorophyll a-b binding protein 8 - chloroplast precursor (unigene aCL247Contig1)	-2.51	**-3.33**	-0.82	-1.23	**-4.42**	**-3.19**	0.34	1.62	-0.75
AT2G05100_Chlorophyll a-b binding protein 151 - chloroplast precursor (unigene aCL5Contig27)	-1.33	-1.60	-0.27	-2.72	**-4.28**	-1.56	1.13	-0.26	-1.55
AT1G15820_Chlorophyll a/b-binding protein CP24 (unigene aCL1214Contig1)	-1.41	**-2.74**	-1.33	-0.73	**-3.89**	**-3.16**	0.98	1.67	-0.17
AT3G47470_Chlorophyll a/b-binding protein (unigene aCL2238Contig1)	-1.91	**-2.45**	-0.54	-1.38	**-3.35**	**-1.98**	0.88	1.42	-0.02
ATCG00350_Photosystem I P700 chlorophyll a apoprotein A1 (unigene aIC0AAA14DF04RM1_c)	-0.32	-1.10	-0.78	-2.88	**-2.60**	0.27	0.82	-1.74	-0.69
ATCG00280_Photosystem II 44 kDa reaction center protein precursor (unigene aKN0AAQ3YK09RM1_c)	0.63	**-1.62**	**-2.25**	-2.58	**-2.15**	0.43	0.68	-2.53	0.15
ATCG00350_Photosystem I P700 chlorophyll a apoprotein A1 (unigene aIC0AAA71CD11RM1_c)	-0.23	-1.19	-0.96	-1.80	**-2.02**	-0.22	0.75	-0.81	-0.07
ATCG00350_Photosystem I P700 chlorophyll a apoprotein A1 (unigene aIC0AAA27CH09RM1_c)	0.91	-0.93	-1.84	-0.53	**-1.90**	-1.37	0.78	-0.65	-0.18
AT3G61470_Chlorophyll a-b binding protein 7 - chloroplast precursor (unigene aCL242Contig1)	-0.97	-1.27	-0.30	-0.09	**-1.82**	**-1.73**	0.29	1.17	-0.26
AT1G44575_Photosystem II 22 kDa protein - chloroplast precursor (unigene aCL1349Contig1)	0.79	-0.83	-1.62	0.59	**-1.66**	**-2.25**	0.12	-0.08	-0.71
AT3G22840_chlorophyll A-B binding family protein / early light-induced protein (EL... (unigene aC18017H11Rv_c)	0.59	1.10	0.51	1.08	**-0.74**	-1.82	0.15	0.64	**-1.69**

**light-harvesting complex (GO:0030076)**	**Level 6**	**CC**							
#name	Log_2 (BKN / MGN)	Log_2 (FCN / MGN)	Log_2 (FCN / BKN)	Log_2 (BKP / MGP)	Log_2 (FCP / MGP)	Log_2 (FCP / BKP)	Log_2 (MGP / MGN)	Log_2 (BKP / BKN)	Log_2 (FCP / FCN)
AT5G01530_Lhcb4-protein (unigene aCL397Contig1)	-1.45	**-4.03**	-2.58	-1.09	**-5.81**	**-4.72**	1.10	1.46	-0.68
AT5G54270_LHCII type III chlorophyll a/b binding protein (unigene aC31707D09EF_c)	-1.23	**-2.51**	-1.28	-1.46	**-4.68**	-3.23	1.19	0.96	-0.99
AT1G29910_Chlorophyll a/b-binding protein (unigene aCL14Contig7)	-0.86	**-2.59**	-1.73	-0.72	**-4.63**	**-3.92**	0.85	1.00	-1.19
AT4G10340_Type I (26 kD) CP29 polypeptide (unigene aCL93Contig2)	**-1.45**	-3.31	-1.85	-2.17	**-4.50**	-2.33	0.86	0.15	-0.33
AT1G61520_Chlorophyll a-b binding protein 8 - chloroplast precursor (unigene aCL247Contig1)	-2.51	**-3.33**	-0.82	-1.23	**-4.42**	**-3.19**	0.34	1.62	-0.75
AT2G05100_Chlorophyll a-b binding protein 151 - chloroplast precursor (unigene aCL5Contig27)	-1.33	-1.60	-0.27	-2.72	**-4.28**	-1.56	1.13	-0.26	-1.55
AT3G47470_Chlorophyll a/b-binding protein (unigene aCL2238Contig1)	-1.91	**-2.45**	-0.54	-1.38	**-3.35**	**-1.98**	0.88	1.42	-0.02
AT3G61470_Chlorophyll a-b binding protein 7 - chloroplast precursor (unigene aCL242Contig1)	-0.97	-1.27	-0.30	-0.09	**-1.82**	**-1.73**	0.29	1.17	-0.26
AT1G44575_Photosystem II 22 kDa protein - chloroplast precursor (unigene aCL1349Contig1)	0.79	-0.83	-1.62	0.59	**-1.66**	**-2.25**	0.12	-0.08	-0.71

Table S2 (cont.)

Photosynthesis (ath00195)	KEGG								
#name	Log_2 (BKN / MGN)	Log_2 (FCN / MGN)	Log_2 (FCN / BKN)	Log_2 (BKP / MGP)	Log_2 (FCP / MGP)	Log_2 (FCP / BKP)	Log_2 (MGP / MGN)	Log_2 (BKP / BKN)	Log_2 (FCP / FCN)
AT1G31330_At1g31330/T19E23_1 (unigene aCL329Contig5)	0.45	-1.14	-1.59	-0.06	**-4.54**	**-4.48**	0.82	0.30	**-2.59**
AT1G52230_Photosystem I reaction center subunit VI - chloroplast precursor (unigene aCL2388Contig1)	-0.05	-1.39	-1.34	-1.25	**-4.27**	**-3.01**	0.73	-0.47	**-2.14**
AT5G64040_Photosystem I reaction centre subunit N - chloroplast precursor (unigene aCL1971Contig1)	-1.05	-1.45	-0.40	-1.01	**-3.99**	**-2.98**	0.72	0.75	-1.83
AT1G55670_Photosystem I reaction center subunit V - chloroplast precursor (unigene aCL223Contig1)	-0.80	-1.25	-0.45	-0.70	**-3.88**	**-3.17**	0.55	0.65	**-2.08**
AT4G03280_Cytochrome b6-f complex iron-sulfur subunit - chloroplast precursor (unigene aCL1340Contig1)	0.29	**-1.48**	-1.77	-0.54	**-3.85**	**-3.31**	0.33	-0.50	-2.03
AT4G12800_Photosystem I subunit XI (unigene aCL2872Contig1)	0.45	-1.42	**-1.87**	0.65	**-3.71**	**-4.35**	0.53	0.73	**-1.75**
AT1G60950_Ferredoxin - chloroplast precursor (unigene aCL6164Contig1)	0.30	-1.30	-1.60	-0.15	**-3.43**	**-3.28**	0.01	-0.45	**-2.12**
AT1G67740_Photosystem II core complex proteins psbY - chloroplast precursor (L- arg... (unigene aCL1522Contig1)	-0.93	-3.66	-2.73	-0.57	**-3.33**	**-2.76**	0.33	0.70	0.66
AT1G79040_Chloroplast photosystem II 10 kDa protein (unigene aCL148Contig1)	-0.16	**-1.54**	-1.38	0.28	**-3.27**	**-3.55**	0.34	0.78	**-1.39**
AT4G03280_Cytochrome b6-f complex iron-sulfur subunit - chloroplast precursor (unigene aC31006G07EF_c)	0.47	-1.15	-1.62	0.02	**-2.84**	**-2.86**	0.68	0.23	-1.01
AT2G30570_Photosystem II reaction center W protein - chloroplast precursor (unigene aCL1139Contig1)	0.41	-0.14	-0.56	-0.40	**-2.74**	**-2.33**	0.49	-0.33	**-2.10**
AT4G02770_Photosystem I reaction center subunit II - chloroplast precursor (unigene aCL3140Contig1)	-0.74	-2.66	-1.93	0.53	**-2.30**	**-2.83**	0.35	1.62	0.71
AT4G09650_Putative H+-transporting ATP synthase (unigene aCL2620Contig1)	0.14	-0.59	-0.73	0.48	**-2.05**	**-2.53**	0.24	0.59	-1.22
AT1G44575_Photosystem II 22 kDa protein - chloroplast precursor (unigene aCL1349Contig1)	0.79	-0.83	-1.62	0.59	**-1.66**	**-2.25**	0.12	-0.08	-0.71
AT5G66190_Ferredoxin--NADP reductase - chloroplast precursor (unigene aCL2953Contig1)	-0.48	0.01	0.49	1.55	**-1.13**	**-2.68**	0.07	2.09	**-1.07**

Photosynthesis antenna-proteins (ath00196)	KEGG								
#name	Log_2 (BKN / MGN)	Log_2 (FCN / MGN)	Log_2 (FCN / BKN)	Log_2 (BKP / MGP)	Log_2 (FCP / MGP)	Log_2 (FCP / BKP)	Log_2 (MGP / MGN)	Log_2 (BKP / BKN)	Log_2 (FCP / FCN)
AT5G01530_Lhcb4-protein (unigene aCL397Contig1)	-1.45	**-4.03**	-2.58	-1.09	**-5.81**	**-4.72**	1.10	1.46	-0.68
AT5G54270_LHCII type III chlorophyll a/b binding protein (unigene aC31707D09EF_c)	-1.23	**-2.51**	-1.28	-1.46	**-4.68**	-3.23	1.19	0.96	-0.99
AT1G29910_Chlorophyll a/b-binding protein (unigene aCL14Contig7)	-0.86	**-2.59**	-1.73	-0.72	**-4.63**	**-3.92**	0.85	1.00	-1.19
AT4G10340_Type I (26 kD) CP29 polypeptide (unigene aCL93Contig2)	**-1.45**	-3.31	-1.85	-2.17	**-4.50**	-2.33	0.86	0.15	-0.33
AT1G61520_Chlorophyll a-b binding protein 8 - chloroplast precursor (unigene aCL247Contig1)	-2.51	**-3.33**	-0.82	-1.23	**-4.42**	**-3.19**	0.34	1.62	-0.75
AT2G05100_Chlorophyll a-b binding protein 151 - chloroplast precursor (unigene aCL5Contig27)	-1.33	-1.60	-0.27	-2.72	**-4.28**	-1.56	1.13	-0.26	-1.55
AT1G15820_Chlorophyll a/b-binding protein CP24 (unigene aCL1214Contig1)	-1.41	**-2.74**	-1.33	-0.73	**-3.89**	**-3.16**	0.98	1.67	-0.17
AT3G47470_Chlorophyll a/b-binding protein (unigene aCL2238Contig1)	-1.91	**-2.45**	-0.54	-1.38	**-3.35**	**-1.98**	0.88	1.42	-0.02
AT3G61470_Chlorophyll a-b binding protein 7 - chloroplast precursor (unigene aCL242Contig1)	-0.97	-1.27	-0.30	-0.09	**-1.82**	**-1.73**	0.29	1.17	-0.26

Carbon fixation in photosynthetic organisms (ath00710)	KEGG								
#name	Log_2 (BKN / MGN)	Log_2 (FCN / MGN)	Log_2 (FCN / BKN)	Log_2 (BKP / MGP)	Log_2 (FCP / MGP)	Log_2 (FCP / BKP)	Log_2 (MGP / MGN)	Log_2 (BKP / BKN)	Log_2 (FCP / FCN)
AT5G38410_Ribulose bisphosphate carboxylase small chain - chloroplast precursor (unigene aCL43Contig3)	-0.89	**-2.78**	-1.88	-1.85	**-4.56**	**-2.72**	0.54	-0.42	-1.25
AT4G38970_Plastidic aldolase (unigene aCL73Contig1)	-0.61	**-2.39**	-1.78	-0.85	**-3.67**	**-2.82**	0.52	0.27	-0.77
AT1G32060_Phosphoribulokinase - chloroplast precursor (unigene aCL1319Contig2)	0.19	-0.61	-0.80	-0.06	**-2.09**	**-2.03**	0.27	0.02	**-1.20**
AT1G23310_F26F24.16 (unigene aCL3840Contig1)	0.14	-0.58	-0.72	0.35	**-1.67**	-2.03	0.69	0.90	-0.41
AT2G19900_NADP-dependent malic enzyme (unigene aCL158Contig1)	-0.32	-0.13	0.19	0.07	**-1.60**	**-1.67**	0.28	0.67	-1.19
AT3G60750_Transketolase-like protein (unigene aCL6739Contig1)	-1.14	-1.27	-0.13	-9.00	**-1.56**	9.00	0.09	-9.00	-0.20
AT1G70580_AlaT1 (unigene aCL7069Contig1)	-0.02	-0.79	-0.77	0.90	**-1.54**	-2.44	0.29	1.21	-0.46
AT2G45290_Transketolase - chloroplast (unigene aCL2018Contig1)	-0.59	-0.90	-0.31	-0.49	**-1.47**	**-0.98**	0.23	0.34	-0.33
AT2G22780_Malate dehydrogenase - glyoxysomal precursor (unigene aCL1958Contig1)	-0.21	-1.05	-0.85	0.70	**-1.25**	**-1.95**	0.35	1.26	0.16
AT1G53310_Phosphoenolpyruvate carboxylase - housekeeping isozyme (unigene aCL6459Contig1)	-0.19	-0.65	-0.46	-1.11	**-1.14**	-0.02	-0.08	-0.99	-0.56
AT5G61410_Ribulose-5-phosphate-3-epimerase (unigene aCL2443Contig1)	0.07	-0.74	-0.81	-0.18	**-1.13**	-0.95	0.13	-0.12	-0.26
AT2G30970_Aspartate aminotransferase - mitochondrial precursor (unigene aKN0AAI2DE02FM1_c)	-0.29	-0.66	-0.37	0.49	**-0.99**	-1.48	0.16	0.94	-0.17
AT1G56190_Phosphoglycerate kinase - chloroplast precursor (unigene aCL955Contig2)	-0.37	-0.60	-0.22	-1.01	**-0.67**	0.34	0.08	-0.56	0.00

Flavonoid biosynthesis (ath00941)	KEGG								
#name	Log_2 (BKN / MGN)	Log_2 (FCN / MGN)	Log_2 (FCN / BKN)	Log_2 (BKP / MGP)	Log_2 (FCP / MGP)	Log_2 (FCP / BKP)	Log_2 (MGP / MGN)	Log_2 (BKP / BKN)	Log_2 (FCP / FCN)
AT5G54160_Eugenol O-methyltransferase (unigene aCL38Contig8)	**-3.62**	**-5.45**	-1.82	**-4.01**	**-6.74**	**-2.73**	0.73	0.34	-0.56
AT5G13930_Chalcone synthase 1 (unigene aCL27Contig2)	-1.37	0.01	1.37	-1.54	**-2.64**	-1.10	-0.04	-0.22	**-2.69**
AT5G13930_Acridone synthase II (unigene aC31807C06EF_c)	-1.25	-0.78	0.46	-0.50	**-2.30**	**-1.80**	0.58	1.33	-0.93
AT5G54160_Eugenol O-methyltransferase (unigene aC31207A03EF_c)	-0.92	-2.95	-2.04	-0.24	**-2.17**	**-1.93**	-0.07	0.60	0.71
AT5G13930_Chalcone synthase (unigene aC31802G12EF_c)	-1.07	-0.81	0.26	0.57	**-2.05**	**-2.61**	0.46	2.09	-0.79
AT5G54160_Eugenol O-methyltransferase (unigene aC31705B10EF_c)	-1.28	-1.72	-0.44	0.13	**-2.03**	**-2.16**	-0.05	1.36	-0.36
AT5G54160_Caffeic acid 3-O-methyltransferase (unigene aCL3343Contig1)	-0.65	-0.81	-0.16	-1.02	**-1.20**	-0.18	0.57	0.20	0.18
AT5G07990_Cytochrome P450 DDWF1 (unigene aCL9318Contig1)	-0.47	-0.65	-0.17	-0.12	**-0.88**	-0.76	0.13	0.48	-0.11

Figure S1. ABA accumulation, color evolution, and maturity index of Navelate (black) and Pinalate (white) fruit along ripening. Results are the means of three biological replicates of 5 fruits each ±SE. Asterisks indicate statistical differences between genotypes according to a *t*-test (*pvalue*<0.05) for each ripening stage.

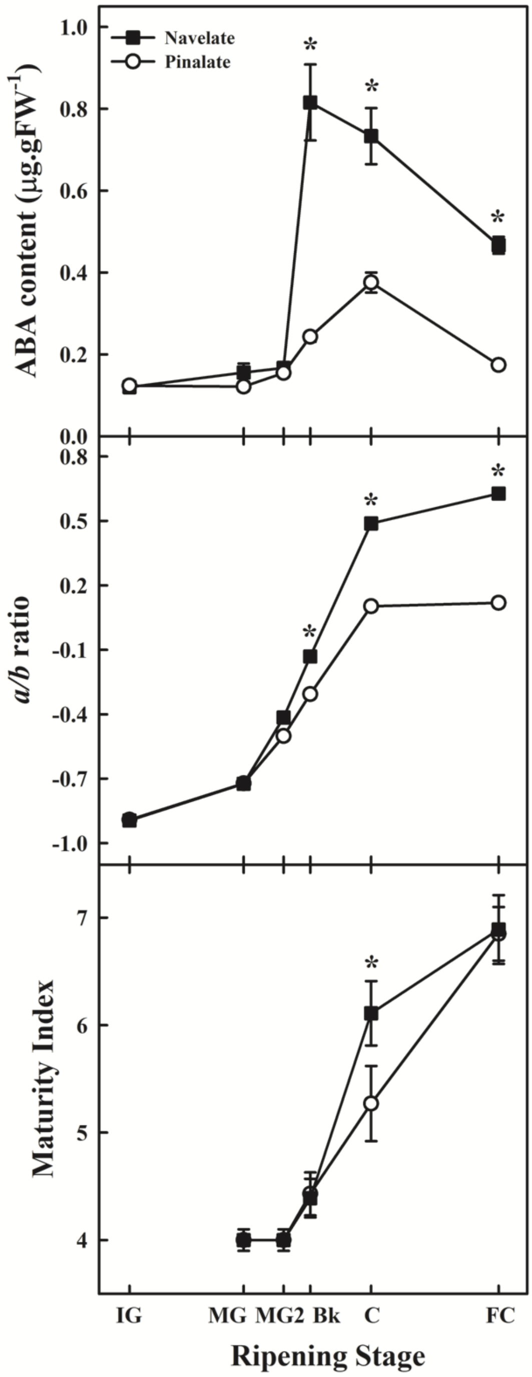

Figure S2. Multiple linear regression analysis (R^2) for the comparison between the Citrus 20K microarray and qRT-PCR gene expression data for the genes listed on Supplemental Table S1.

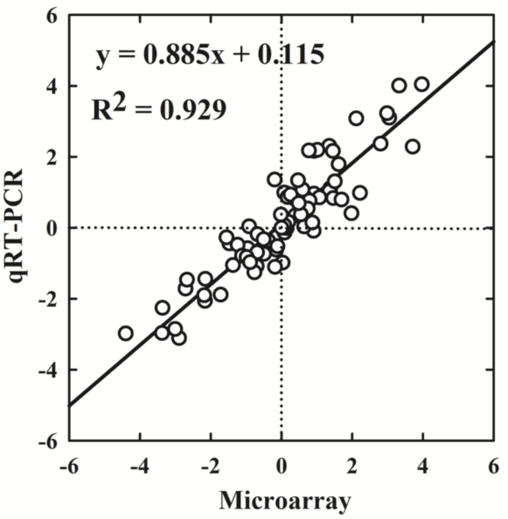

Supplement: Supplementary file 1 — Supplementary Information [file 41598_2019_46365_MOESM1_ESM.pdf]
